# Supplementary figures and images for: Identification and Evolutionary Analysis of the Auxin Response Factor (ARF) Family Based on Transcriptome Data from Caucasian Clover and Analysis of Expression Responses to Hormones
Source: Int J Mol Sci. 2023 Oct 19;24(20):15357. doi: 10.3390/ijms242015357 (PMC10607010; doi:10.3390/ijms242015357)

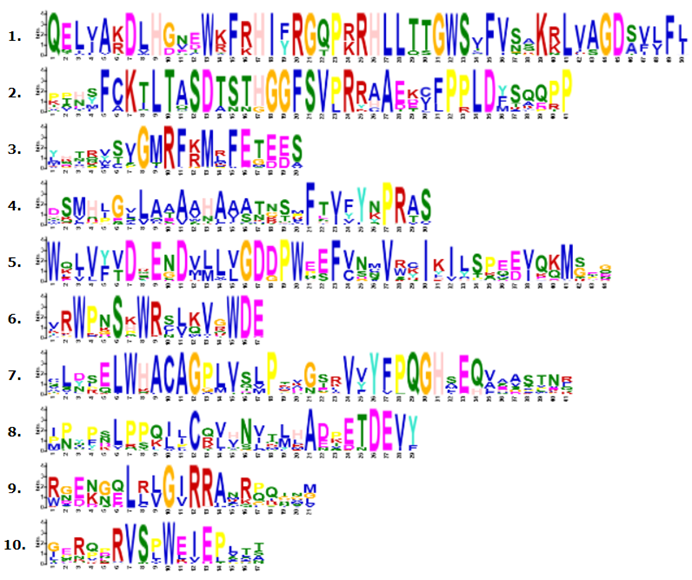

Supplement: Supplementary file 1 [file ijms-24-15357-s001.zip › Figure S1.png]
